# Supplementary material for: Potomania and Beer Potomania: A Systematic Review of Published Case Reports
Source: Nutrients. 2025 Jun 16;17(12):2012. doi: 10.3390/nu17122012 (PMC12196016; doi:10.3390/nu17122012)
Supplement: Supplementary file 1 [file nutrients-17-02012-s001.zip › nutrients-3670251-supplementary.pdf]

**Table S1.** JBI critical appraisal checklist for case reports.

| **Author and Year** | **Were patient's demographic characteristics clearly described?** | **Was the patient's history clearly described and presented as a timeline?** | **Was the current clinical condition of the patient on presentation clearly described?** | **Were diagnostic tests or assessment methods and the results clearly described?** | **Was the intervention[s] or treatment procedure[s] clearly described?** | **Was the post-intervention clinical condition clearly described?** | **Were adverse events [harms] or unanticipated events identified and described?** | **Does the case report provide takeaway lessons?** |
|---|---|---|---|---|---|---|---|---|
| Lodhi et al. [2017] | Yes | Yes | Yes | Yes | Yes | Yes | Yes | No |
| Yu ZL and Fisher [2022] | Yes | Yes | Yes | Yes | Yes | Yes | Yes | No |
| Bhattarai et al. [2010] | Yes | Yes | Yes | Yes | Yes | Yes | Yes | Yes |
| Kujubu et al. [2015] | Yes | Yes | Yes | Yes | Yes | Yes | Yes | Yes |
| Senanayake et al. [2023] | Yes | Yes | Yes | Yes | Yes | Yes | Yes | Yes |
| Dodoo et al. [2022] | Yes | Yes | Yes | Yes | Yes | Yes | Yes | No |
| Stasishin et al. [2021] | Unclear | Yes | Yes | Yes | Yes | Yes | Yes | Yes |
| Laubner et al. [2020] | Yes | Yes | Yes | Yes | Yes | No | Yes | No |

| | | | | | | | | |
|---|---|---|---|---|---|---|---|---|
| Mifsud et al. [2018] | Yes | Yes | Yes | Yes | Yes | No | Yes | Yes |
| So BYF and Chan GCW [2023] | Yes | Yes | Yes | Yes | Yes | No | Yes | No |
| Perez Gomez et al. [2022] | Yes | Unclear | Yes | Unclear | No | No | No | Yes |
| Macías Robles et al. [2009] | Yes | Yes | Yes | Yes | Yes | No | Yes | No |
| Soliman et al. [2018] | Yes | Yes | Yes | Yes | Yes | Unclear | Yes | No |
| Sterns et al. [2010] | Yes | Yes | Yes | Yes | Yes | Yes | Yes | Yes |
| Lum G. [2013] | Yes | Yes | Yes | Yes | Yes | No | No | Yes |
| Windpessl et al. [2017] | Yes | Yes | Yes | Yes | Yes | No | No | No |
| Rafei et al. [2016] | Yes | Yes | Yes | Yes | Unclear | No | No | No |
| Pallavi, R. [2015] | Yes | Yes | Yes | Yes | Yes | No | Yes | No |
| Srisung et al. [2015] | Yes | Yes | Yes | Yes | Yes | Yes | No | No |
| Patel et al. [2012] | Yes | Yes | Yes | Yes | Unclear | No | Yes | No |

| | | | | | | | | |
|---|---|---|---|---|---|---|---|---|
| McGraw et al. [2012] | Yes | Yes | Yes | Yes | Yes | No | Yes | Yes |
| Haran et al. [2010] | Yes | Yes | Yes | Yes | Yes | No | Yes | No |
| Fox BD [2002] | Yes | Yes | Yes | Yes | Yes | Yes | Unclear | No |
| Lord [2000] | Yes | Yes | Yes | Yes | Yes | No | No | No |
| Sharif et al. [2023] | Yes | Yes | Yes | Unclear | Unclear | No | No | Unclear |
| Akatsuka et al. [2023] | Yes | Yes | Yes | Yes | Yes | No | Yes | No |
| Campeão [2013] | Yes | Unclear | Yes | No | Unclear | No | No | No |
| Chaudhary et al. [2012] | Yes | Yes | Yes | Yes | Yes | No | Yes | No |
| Axelrod et al. [2011] | Yes | Yes | Yes | No | Yes | No | No | No |
| Pizzini et al. [2011] | Yes | Yes | Unclear | No | Unclear | No | No | No |
| Caron et al. [1977] | Yes | Yes | Yes | Yes | Yes | No | No | No |
| Goëb et al. [2003] | Yes | Yes | Yes | No | No | Yes | No | No |
| Hirel and Thobois [2014] | Yes | Yes | Yes | Unclear | Yes | No | No | No |

| | | | | | | | | |
|---|---|---|---|---|---|---|---|---|
| Couillard et al. [2021] | Yes | Yes | Yes | Yes | Yes | Yes | No | No |
| Haymann [2014] | Yes | Yes | Yes | Yes | Yes | No | No | No |
| Tejedor et al. [2014] | Yes | Yes | Yes | Yes | Yes | No | No | No |
| Domínguez et al. [2013] | Yes | Yes | Yes | Yes | Unclear | Unclear | Yes | No |
| Alda et al. [2007] | Yes | Yes | Yes | Unclear | No | No | No | No |
| Benítez-Mejía et al. [2021] | Yes | Yes | Yes | Yes | Yes | No | No | No |
| Milisenda et al. [2012] | Yes | No | Yes | Yes | Yes | Yes | No | No |
| Campbell [2010] | Yes | Yes | Yes | Yes | Yes | Unclear | Unclear | No |

| | | | | | | | | |
|---|---|---|---|---|---|---|---|---|
| **Total** | 40 | 38 | 40 | 33 | 33 | 13 | 20 | 9 |
| | 1 | 2 | 1 | 4 | 6 | 3 | 2 | 1 |
| | 0 | 1 | 0 | 4 | 2 | 25 | 19 | 31 |

**Table S2.** PRISMA 2020 Checklist.

| Section and Topic | Item # | Checklist item | Location where item is reported |
|---|---|---|---|
| **TITLE: Potomania: Systematic Review: Case Reports of Potomania** | | | |
| Title | 1 | Identify the report as a systematic review | Line 1 |
| **ABSTRACT** | | | |
| Abstract | 2 | See the PRISMA 2020 for Abstracts checklist. | Line 13 - 38 |
| **INTRODUCTION** | | | |
| Rationale | 3 | Describe the rationale for the review in the context of existing knowledge. | Line 73 - 118 |
| Objectives | 4 | Provide an explicit statement of the objective(s) or question(s) the review addresses. | Line 120-123 |
| **METHODS** | | | |
| Eligibility criteria | 5 | Specify the inclusion and exclusion criteria for the review and how studies were grouped for the syntheses. | Line 35 - 148 |
| Information sources | 6 | Specify all databases, registers, websites, organisations, reference lists and other sources searched or consulted to identify studies. Specify the date when each source was last searched or consulted. | Line 128 - 132 |
| Search strategy | 7 | Present the full search strategies for all databases, registers and websites, including any filters and limits used. | Line 132 - 134 |
| Selection process | 8 | Specify the methods used to decide whether a study met the inclusion criteria of the review, including how many reviewers screened each record and each report retrieved, whether they worked independently, and if applicable, details of automation tools used in the process. | Line 149 - 153 |
| Data collection process | 9 | Specify the methods used to collect data from reports, including how many reviewers collected data from each report, whether they worked independently, any processes for obtaining or confirming data from study investigators, and if applicable, details of automation tools used in the process. | Line 149 - 153 |
| Data items | 10a | List and define all outcomes for which data were sought. Specify whether all results that were compatible with each outcome domain in each study were sought (e.g. for all measures, time points, analyses), and if not, the methods used to decide which results to collect. | Line 147 |
| | 10b | List and define all other variables for which data were sought (e.g. participant and intervention characteristics, funding sources). Describe any assumptions made about any missing or unclear information. | Line 147 |
| Study risk of bias assessment | 11 | Specify the methods used to assess risk of bias in the included studies, including details of the tool(s) used, how many reviewers assessed each study and whether they worked independently, and if applicable, details of automation tools used in the process. | Line 154 - 157 |
| Effect measures | 12 | Specify for each outcome the effect measure(s) (e.g. risk ratio, mean difference) used in the synthesis or presentation of results. | Not applicable |
| Synthesis methods | 13a | Describe the processes used to decide which studies were eligible for each synthesis (e.g. tabulating the study intervention characteristics and comparing against the planned groups for each synthesis (item #5)). | Line 158 - 159 |
| | 13b | Describe any methods required to prepare the data for presentation or synthesis, such as handling of missing summary statistics, or data conversions. | Not applicable |
| | 13c | Describe any methods used to tabulate or visually display results of individual studies and syntheses. | Not applicable |

| Section and Topic | Item # | Checklist item | Location where item is reported |
|---|---|---|---|
| | 13d | Describe any methods used to synthesize results and provide a rationale for the choice(s). If meta-analysis was performed, describe the model(s), method(s) to identify the presence and extent of statistical heterogeneity, and software package(s) used. | Not applicable |
| | 13e | Describe any methods used to explore possible causes of heterogeneity among study results (e.g. subgroup analysis, meta-regression). | Not applicable |
| | 13f | Describe any sensitivity analyses conducted to assess robustness of the synthesized results. | Not applicable |
| Reporting bias assessment | 14 | Describe any methods used to assess risk of bias due to missing results in a synthesis (arising from reporting biases). | Not applicable |
| Certainty assessment | 15 | Describe any methods used to assess certainty (or confidence) in the body of evidence for an outcome. | Not applicable |
| **RESULTS** | | | |
| Study selection | 16a | Describe the results of the search and selection process, from the number of records identified in the search to the number of studies included in the review, ideally using a flow diagram. | Line 165 – 178<br>Figure 1 |
| | 16b | Cite studies that might appear to meet the inclusion criteria, but which were excluded, and explain why they were excluded. | Line 175 - 178 |
| Study characteristics | 17 | Cite each included study and present its characteristics. | Table 2 – 4 line 194 |
| Risk of bias in studies | 18 | Present assessments of risk of bias for each included study. | Line 191 - 193 |
| Results of individual studies | 19 | For all outcomes, present, for each study: (a) summary statistics for each group (where appropriate) and (b) an effect estimate and its precision (e.g. confidence/credible interval), ideally using structured tables or plots. | Table 3 – 4 Line 181 - 193 |
| Results of syntheses | 20a | For each synthesis, briefly summarise the characteristics and risk of bias among contributing studies. | Line 179-193 |
| | 20b | Present results of all statistical syntheses conducted. If meta-analysis was done, present for each the summary estimate and its precision (e.g. confidence/credible interval) and measures of statistical heterogeneity. If comparing groups, describe the direction of the effect. | Not applicable |
| | 20c | Present results of all investigations of possible causes of heterogeneity among study results. | Not applicable |
| | 20d | Present results of all sensitivity analyses conducted to assess the robustness of the synthesized results. | Not applicable |
| Reporting biases | 21 | Present assessments of risk of bias due to missing results (arising from reporting biases) for each synthesis assessed. | Not applicable |
| Certainty of evidence | 22 | Present assessments of certainty (or confidence) in the body of evidence for each outcome assessed. | Not applicable |
| **DISCUSSION** | | | |
| Discussion | 23a | Provide a general interpretation of the results in the context of other evidence. | Line 263 - 280 |

| Section and Topic | Item # | Checklist item | Location where item is reported |
|---|---|---|---|
| | 23b | Discuss any limitations of the evidence included in the review. | Line 310-315 |
| | 23c | Discuss any limitations of the review processes used. | Line 315 |
| | 23d | Discuss implications of the results for practice, policy, and future research. | Line 316 - 321 |
| **OTHER INFORMATION** | | | |
| Registration and protocol | 24a | Provide registration information for the review, including register name and registration number, or state that the review was not registered. | Line 126 |
| | 24b | Indicate where the review protocol can be accessed, or state that a protocol was not prepared. | Line 126 |
| | 24c | Describe and explain any amendments to information provided at registration or in the protocol. | Not applicable |
| Support | 25 | Describe sources of financial or non-financial support for the review, and the role of the funders or sponsors in the review. | Line 329 |
| Competing interests | 26 | Declare any competing interests of review authors. | Line 332 |
| Availability of data, code and other materials | 27 | Report which of the following are publicly available and where they can be found: template data collection forms; data extracted from included studies; data used for all analyses; analytic code; any other materials used in the review. | Line 331 |

*From:* Page MJ, McKenzie JE, Bossuyt PM, Boutron I, Hoffmann TC, Mulrow CD, et al. The PRISMA 2020 statement: an updated guideline for reporting systematic reviews. BMJ 2021;372:n71. doi: 10.1136/bmj.n71
